# (η^5^-Cyclo­penta­dien­yl)[η^6^-diethyl eth­yl(phen­yl)malonate]iron(II) hexa­fluorido­phosphate

**DOI:** 10.1107/S1600536808030523

**Published:** 2008-10-04

**Authors:** Hilary A. Jenkins, Jason D. Masuda, Adam Piórko

**Affiliations:** a Department of Chemistry, Saint Mary’s University, Halifax, Nova Scotia, Canada B3H 3C3

## Abstract

At 223 (2) K, the complexed rings in the iron(II) complex cation of the title salt, [Fe(C_5_H_5_)(C_15_H_20_O_4_)]PF_6_, are almost parallel [dihedral angle between planes = 4.10 (14)°]. Among the C atoms of the complexed arene ring, the quaternary C atom is located at the longest distance from the Fe atom. The F atoms of the PF_6_^−^ anion were found to be equally disordered over two sites.

## Related literature

For related literature, see: Abboud *et al.* (1991 ▶); Crane (2003 ▶); Hanson (1982 ▶); Koray *et al.* (1985 ▶); Marcén *et al.* (2002 ▶); Piórko *et al.* (1989 ▶, 1994 ▶).
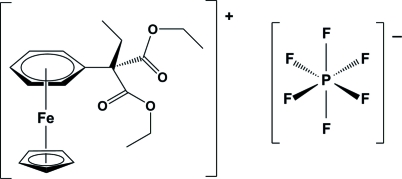


## Experimental

### 

#### Crystal data

[Fe(C_5_H_5_)(C_15_H_20_O_4_)]PF_6_
*M* *_r_* = 530.22
Triclinic, 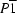

*a* = 10.1075 (5) Å
*b* = 10.6731 (5) Å
*c* = 11.4797 (6) Å
α = 100.396 (1)°
β = 111.854 (1)°
γ = 99.100 (1)°
*V* = 1095.56 (9) Å^3^
*Z* = 2
Mo *K*α radiation
μ = 0.84 mm^−1^
*T* = 223 (2) K
0.40 × 0.38 × 0.10 mm

#### Data collection

Bruker SMART CCD area-detector diffractometer
Absorption correction: multi-scan (*SADABS*; Bruker, 1998 ▶) *T*_min_ = 0.688, *T*_max_ = 0.920
8715 measured reflections
4253 independent reflections
3283 reflections with *I* > 2σ(*I*)
*R*_int_ = 0.016

#### Refinement

*R*[*F* ^2^ > 2σ(*F* ^2^)] = 0.029
*wR*(*F* ^2^) = 0.070
*S* = 0.95
4253 reflections
343 parameters
30 restraints
H-atom parameters constrained
Δρ_max_ = 0.27 e Å^−3^
Δρ_min_ = −0.26 e Å^−3^

### 

Data collection: *SMART* (Bruker, 1998 ▶); cell refinement: *SAINT* (Bruker, 1998 ▶); data reduction: *SAINT* and *SHELXTL* (Sheldrick, 2008 ▶); program(s) used to solve structure: *SHELXS97* (Sheldrick, 2008 ▶); program(s) used to refine structure: *SHELXL97* (Sheldrick, 2008 ▶); molecular graphics: *SHELXTL*; software used to prepare material for publication: *SHELXTL*.

## Supplementary Material

Crystal structure: contains datablocks I, global. DOI: 10.1107/S1600536808030523/rt2022sup1.cif

Structure factors: contains datablocks I. DOI: 10.1107/S1600536808030523/rt2022Isup2.hkl

Additional supplementary materials:  crystallographic information; 3D view; checkCIF report

## Figures and Tables

**Table 1:** Selected geometric parameters (Å)

Fe1—C15	2.072 (2)
Fe1—C16	2.073 (2)
Fe1—C13	2.077 (2)
Fe1—C14	2.078 (2)
Fe1—C12	2.0906 (19)
Fe1—C11	2.1179 (19)
Fe1—C21	2.050 (2)
Fe1—C24	2.050 (2)
Fe1—C25	2.052(2)
Fe1—C23	2.053 (2)
Fe1—C22	2.063 (2)

**Fig. 1.**
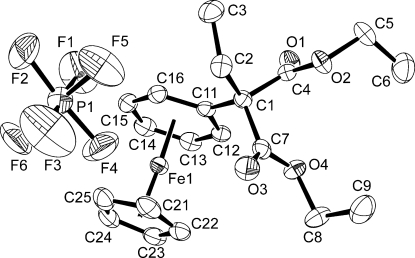
View of the cation and anion showing the labelling of non-H atoms with the thermal ellipsoids shown at 50% probability levels.

**Fig. 2.**
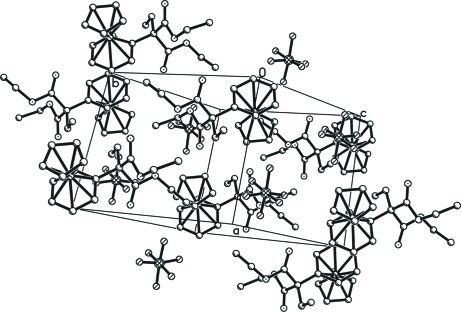
The packing diagram for the molecule.

## supplementary crystallographic information

### Comment

The title compound, along with other diethyl alkylarylmalonates complexed with a cyclopentadienyliron(II) moiety, were reported as intermediates in the syntheses of alkyl (substituted aryl)malonates (Piórko *et al.*, 1989). The *ORTEP* drawing of the title compound is shown in Fig. 1. The two aromatic rings are not quite coplanar, with a dihedral angle of 4.10(0.14)° between the plane formed by C11–C16 and C21–C25 which is not significantly different from the value 3.0 (4)° reported for the most similar structure of 1,1-di(2-propenyl)-3-butenylbenzene FeCp complex (Marcén *et al.*, 2002). The Fe ion is located at distances of 1.6661 (9)Å from the Cp and 1.5360 (8)Å from the phenyl ring; such values are typical of those found in the literature for similar complexes (see for example Abboud *et al.*, 1991; Piórko *et al.*, 1994; Marcén *et al.*, 2002; Crane 2003). The distance for Fe1—C11 at 2.1179 (19) Å, where C11 is the quaternary carbon in the phenyl ring, is the longest among the distances Fe to C atoms of this ring. Again, this is in agreement with the literature data for the similar FeCp complexed aromatic C atoms containing alkyl groups (see for example Hanson 1982; Koray *et al.*, 1985; Piórko *et al.*, 1994; Marcén *et al.*, 2002). The PF_6_^-^ anion shows some rotational disorder, as reported for systems of this kind. A view along a side of the unit cell (Fig. 2) indicates π–π stacking arrangement of the aromatic rings, with a centroid to centroid distance of 3.85 Å. The rings are oriented in a face to face arrangement, and while this almost direct overlap is uncommon for unsubstituted arene rings, the ester functionality on the ring alters the electronics of the system, making it possible for the rings to interact; the functional group on one ring is on the opposite side of that on the next ring.

### Experimental

The title compound was prepared following the method of Piórko *et al.* (1989). A crystal used for data collection was grown by slow evaporation of solvents from a solution of the complex in acetone–diethyl ether–dichloromethane mixture at 280 K.

### Refinement

The H atoms were placed in geometrically idealized positions with C—H distances of 0.93 Å (aromatic), 0.96 Å (methyl) and 0.97 Å (methylene) and constrained to ride on the parent C atom with Uiso(H) = 1.2Ueq(C) for aromatic and methylene, and Uiso(H) = 1.5Ueq(C) for methyl protons. Then F atoms of the PF_6_ anion were found to be disordered over two sites and were refined with a 50:50 occupancy. The F atoms were anisotropically refined and F1, F2, F3, F4 and F4' restrained using the ISOR command.

### Crystal data

**Table:**

[Fe(C_5_H_5_)(C_15_H_20_O_4_)]PF_6_	*Z* = 2
*M**_r_* = 530.22	*F*(000) = 544
Triclinic, *P*1	*D*_x_ = 1.607 Mg m^−^^3^
Hall symbol: -P 1	Mo *K*α radiation, λ = 0.71073 Å
*a* = 10.1075 (5) Å	Cell parameters from 3863 reflections
*b* = 10.6731 (5) Å	θ = 2.2–28.0°
*c* = 11.4797 (6) Å	µ = 0.84 mm^−^^1^
α = 100.396 (1)°	*T* = 223 K
β = 111.854 (1)°	Plate, yellow
γ = 99.100 (1)°	0.40 × 0.38 × 0.10 mm
*V* = 1095.56 (9) Å^3^	

### Data collection

**Table:**

Bruker SMART CCD area-detector diffractometer	4253 independent reflections
Radiation source: fine-focus sealed tube	3283 reflections with *I* > 2σ(*I*)
graphite	*R*_int_ = 0.016
φ and ω scans	θ_max_ = 26.0°, θ_min_ = 2.0°
Absorption correction: multi-scan (SADABS; Bruker, 1998)	*h* = −12→12
*T*_min_ = 0.688, *T*_max_ = 0.920	*k* = −13→13
8715 measured reflections	*l* = −14→14

### Refinement

**Table:**

Refinement on *F*^2^	Primary atom site location: structure-invariant direct methods
Least-squares matrix: full	Secondary atom site location: difference Fourier map
*R*[*F*^2^ > 2σ(*F*^2^)] = 0.029	Hydrogen site location: inferred from neighbouring sites
*wR*(*F*^2^) = 0.070	H-atom parameters constrained
*S* = 0.95	*w* = 1/[σ^2^(*F*_o_^2^) + (0.0326*P*)^2^] where *P* = (*F*_o_^2^ + 2*F*_c_^2^)/3
4253 reflections	(Δ/σ)_max_ = 0.001
343 parameters	Δρ_max_ = 0.27 e Å^−^^3^
30 restraints	Δρ_min_ = −0.26 e Å^−^^3^

### Special details

**Table:**

Geometry. All e.s.d.'s are estimated using the full covariance matrix. The cell e.s.d.'s are taken into account individually in the estimation of e.s.d.'s in distances, angles and torsion angles; correlations between e.s.d.'s in cell parameters are only used when they are defined by crystal symmetry. An approximate (isotropic) treatment of cell e.s.d.'s is used for estimating e.s.d.'s involving l.s. planes.
Refinement. Refinement of *F*^2^ against ALL reflections. The weighted *R*-factor *wR* and goodness of fit *S* are based on *F*^2^, conventional *R*-factors *R* are based on *F*, with *F* set to zero for negative *F*^2^. The threshold expression of *F*^2^ > σ(*F*^2^) is used only for calculating *R*-factors(gt) *etc* and is not relevant to the choice of reflections for refinement. *R*-factors based on *F*^2^ are statistically about twice as large as those based on *F*, and *R*- factors based on ALL data will be even larger.

### Fractional atomic coordinates and isotropic or equivalent isotropic displacement parameters (Å^2^)

**Table:**

	*x*	*y*	*z*	*U*_iso_*/*U*_eq_	Occ. (<1)
Fe1	0.81979 (3)	0.78170 (3)	0.69683 (3)	0.02689 (10)	
O1	0.92832 (15)	0.74047 (14)	1.12205 (13)	0.0373 (4)	
O2	0.72028 (15)	0.60360 (14)	1.09549 (13)	0.0346 (3)	
O3	0.53949 (16)	0.50483 (14)	0.74158 (14)	0.0403 (4)	
O4	0.76727 (15)	0.48640 (13)	0.85723 (13)	0.0329 (3)	
C1	0.7101 (2)	0.68946 (18)	0.91843 (18)	0.0254 (4)	
C2	0.5722 (2)	0.7343 (2)	0.9184 (2)	0.0334 (5)	
H2A	0.5192	0.7499	0.8336	0.040*	
H2B	0.5072	0.6627	0.9286	0.040*	
C3	0.6033 (3)	0.8579 (2)	1.0241 (2)	0.0445 (6)	
H3A	0.5114	0.8797	1.0167	0.067*	
H3B	0.6660	0.9301	1.0140	0.067*	
H3C	0.6524	0.8427	1.1088	0.067*	
C4	0.8028 (2)	0.68215 (18)	1.05709 (18)	0.0272 (4)	
C5	0.7890 (2)	0.5919 (2)	1.2283 (2)	0.0407 (6)	
H5A	0.7138	0.5746	1.2618	0.049*	
H5B	0.8601	0.6748	1.2840	0.049*	
C6	0.8652 (3)	0.4835 (3)	1.2322 (3)	0.0620 (7)	
H6A	0.9108	0.4775	1.3211	0.093*	
H6B	0.9401	0.5010	1.1995	0.093*	
H6C	0.7944	0.4012	1.1785	0.093*	
C7	0.6602 (2)	0.54987 (19)	0.82816 (19)	0.0282 (4)	
C8	0.7289 (3)	0.3497 (2)	0.7801 (2)	0.0420 (6)	
H8A	0.8186	0.3242	0.7823	0.050*	
H8B	0.6654	0.3425	0.6894	0.050*	
C9	0.6513 (3)	0.2591 (2)	0.8327 (3)	0.0582 (7)	
H9A	0.6259	0.1697	0.7801	0.087*	
H9B	0.5623	0.2843	0.8302	0.087*	
H9C	0.7152	0.2647	0.9217	0.087*	
C11	0.8062 (2)	0.78164 (18)	0.87652 (17)	0.0249 (4)	
C12	0.9415 (2)	0.75755 (19)	0.87999 (18)	0.0279 (4)	
H12A	0.9593	0.6699	0.8862	0.034*	
C13	1.0319 (2)	0.8405 (2)	0.84251 (19)	0.0336 (5)	
H13A	1.1117	0.8099	0.8248	0.040*	
C14	0.9897 (2)	0.9497 (2)	0.8011 (2)	0.0383 (5)	
H14A	1.0406	0.9946	0.7556	0.046*	
C15	0.8563 (2)	0.9749 (2)	0.7967 (2)	0.0359 (5)	
H15A	0.8151	1.0369	0.7474	0.043*	
C16	0.7640 (2)	0.89045 (19)	0.83243 (19)	0.0309 (5)	
H16A	0.6604	0.8955	0.8072	0.037*	
C21	0.6282 (2)	0.6578 (2)	0.55586 (19)	0.0400 (6)	
H21A	0.5383	0.6247	0.5679	0.048*	
C22	0.7449 (3)	0.5962 (2)	0.57124 (19)	0.0391 (5)	
H22A	0.7514	0.5127	0.5964	0.047*	
C23	0.8518 (3)	0.6756 (2)	0.5459 (2)	0.0400 (5)	
H23A	0.9460	0.6571	0.5494	0.048*	
C24	0.7992 (3)	0.7845 (2)	0.5129 (2)	0.0409 (6)	
H24A	0.8502	0.8562	0.4892	0.049*	
C25	0.6604 (2)	0.7735 (2)	0.5190 (2)	0.0407 (6)	
H25A	0.5973	0.8359	0.5002	0.049*	
P1	0.27016 (7)	0.85459 (6)	0.57865 (6)	0.04155 (16)	
F1	0.4244 (5)	0.9355 (6)	0.6910 (5)	0.1004 (17)	0.50
F2	0.1990 (10)	0.9654 (8)	0.6111 (6)	0.116 (2)	0.50
F3	0.1268 (7)	0.7640 (9)	0.4775 (7)	0.133 (3)	0.50
F4	0.3508 (9)	0.7418 (6)	0.5589 (7)	0.090 (2)	0.50
F5	0.2264 (15)	0.8121 (18)	0.6816 (17)	0.101 (4)	0.50
F6	0.2812 (16)	0.8822 (15)	0.4546 (10)	0.093 (4)	0.50
F1'	0.3536 (9)	0.9989 (5)	0.6502 (5)	0.110 (2)	0.50
F2'	0.1219 (6)	0.9002 (7)	0.5286 (9)	0.124 (3)	0.50
F3'	0.1751 (10)	0.7108 (5)	0.4937 (8)	0.114 (3)	0.50
F4'	0.4077 (7)	0.8045 (9)	0.6140 (8)	0.117 (3)	0.50
F5'	0.2616 (16)	0.8132 (17)	0.7044 (17)	0.094 (4)	0.50
F6'	0.3100 (15)	0.9107 (15)	0.4735 (12)	0.095 (4)	0.50

### Atomic displacement parameters (Å^2^)

**Table:**

	*U*^11^	*U*^22^	*U*^33^	*U*^12^	*U*^13^	*U*^23^
Fe1	0.02888 (17)	0.02541 (16)	0.02465 (16)	0.00237 (12)	0.01130 (13)	0.00567 (12)
O1	0.0332 (8)	0.0393 (9)	0.0293 (8)	−0.0006 (7)	0.0056 (7)	0.0093 (7)
O2	0.0352 (8)	0.0418 (9)	0.0270 (8)	0.0043 (7)	0.0130 (7)	0.0137 (6)
O3	0.0341 (9)	0.0373 (9)	0.0345 (8)	0.0006 (7)	0.0040 (7)	0.0037 (7)
O4	0.0325 (8)	0.0245 (7)	0.0371 (8)	0.0050 (6)	0.0130 (7)	0.0019 (6)
C1	0.0249 (10)	0.0262 (10)	0.0235 (10)	0.0047 (8)	0.0094 (8)	0.0054 (8)
C2	0.0274 (11)	0.0367 (12)	0.0403 (12)	0.0089 (9)	0.0164 (10)	0.0139 (10)
C3	0.0476 (14)	0.0437 (14)	0.0568 (15)	0.0192 (11)	0.0337 (13)	0.0143 (12)
C4	0.0329 (12)	0.0235 (10)	0.0261 (10)	0.0081 (9)	0.0134 (9)	0.0047 (8)
C5	0.0439 (14)	0.0546 (15)	0.0271 (11)	0.0096 (11)	0.0167 (10)	0.0170 (11)
C6	0.0710 (19)	0.0593 (18)	0.0575 (17)	0.0261 (15)	0.0182 (15)	0.0290 (14)
C7	0.0313 (11)	0.0275 (11)	0.0270 (11)	0.0029 (9)	0.0142 (10)	0.0093 (9)
C8	0.0458 (14)	0.0253 (11)	0.0517 (14)	0.0059 (10)	0.0225 (12)	−0.0001 (10)
C9	0.0716 (19)	0.0321 (13)	0.0714 (18)	0.0060 (13)	0.0327 (16)	0.0140 (12)
C11	0.0264 (10)	0.0234 (10)	0.0199 (10)	0.0023 (8)	0.0080 (8)	0.0003 (8)
C12	0.0265 (10)	0.0276 (11)	0.0231 (10)	0.0026 (9)	0.0074 (9)	0.0011 (8)
C13	0.0266 (11)	0.0370 (12)	0.0311 (11)	−0.0005 (9)	0.0115 (9)	0.0025 (9)
C14	0.0428 (13)	0.0317 (12)	0.0343 (12)	−0.0063 (10)	0.0180 (10)	0.0024 (9)
C15	0.0490 (14)	0.0232 (11)	0.0347 (12)	0.0048 (10)	0.0184 (11)	0.0069 (9)
C16	0.0359 (12)	0.0263 (11)	0.0302 (11)	0.0065 (9)	0.0149 (10)	0.0049 (9)
C21	0.0368 (12)	0.0421 (13)	0.0248 (11)	−0.0083 (10)	0.0055 (10)	0.0014 (10)
C22	0.0531 (15)	0.0299 (12)	0.0247 (11)	0.0028 (11)	0.0115 (10)	0.0010 (9)
C23	0.0485 (14)	0.0438 (14)	0.0281 (11)	0.0094 (11)	0.0195 (11)	0.0038 (10)
C24	0.0517 (14)	0.0430 (14)	0.0260 (11)	0.0026 (11)	0.0169 (11)	0.0106 (10)
C25	0.0405 (13)	0.0451 (14)	0.0278 (12)	0.0082 (11)	0.0054 (10)	0.0098 (10)
P1	0.0377 (3)	0.0473 (4)	0.0485 (4)	0.0136 (3)	0.0237 (3)	0.0180 (3)
F1	0.071 (3)	0.096 (4)	0.083 (3)	−0.021 (3)	0.000 (2)	0.010 (3)
F2	0.172 (6)	0.136 (5)	0.119 (4)	0.127 (5)	0.091 (4)	0.072 (4)
F3	0.054 (3)	0.188 (8)	0.092 (3)	−0.041 (4)	0.000 (3)	0.012 (6)
F4	0.135 (6)	0.066 (3)	0.111 (4)	0.054 (3)	0.083 (4)	0.025 (2)
F5	0.093 (6)	0.147 (9)	0.090 (7)	0.001 (5)	0.072 (6)	0.040 (5)
F6	0.160 (9)	0.083 (5)	0.040 (3)	0.023 (5)	0.043 (4)	0.028 (3)
F1'	0.151 (6)	0.071 (3)	0.065 (3)	−0.050 (3)	0.040 (3)	−0.009 (2)
F2'	0.053 (3)	0.114 (5)	0.228 (8)	0.046 (3)	0.057 (4)	0.077 (5)
F3'	0.171 (9)	0.036 (2)	0.119 (5)	0.006 (3)	0.057 (5)	0.007 (2)
F4'	0.067 (4)	0.197 (8)	0.148 (6)	0.084 (4)	0.061 (4)	0.112 (5)
F5'	0.124 (8)	0.140 (9)	0.081 (6)	0.080 (8)	0.068 (6)	0.077 (6)
F6'	0.099 (5)	0.107 (8)	0.129 (8)	0.032 (5)	0.076 (6)	0.081 (7)

### Geometric parameters (Å, °)

**Table:**

Fe1—C21	2.050 (2)	C9—H9A	0.9700
Fe1—C24	2.050 (2)	C9—H9B	0.9700
Fe1—C25	2.052 (2)	C9—H9C	0.9700
Fe1—C23	2.053 (2)	C11—C16	1.413 (3)
Fe1—C22	2.063 (2)	C11—C12	1.418 (3)
Fe1—C15	2.072 (2)	C12—C13	1.406 (3)
Fe1—C16	2.073 (2)	C12—H12A	0.9900
Fe1—C13	2.077 (2)	C13—C14	1.405 (3)
Fe1—C14	2.078 (2)	C13—H13A	0.9900
Fe1—C12	2.0906 (19)	C14—C15	1.400 (3)
Fe1—C11	2.1179 (19)	C14—H14A	0.9900
O1—C4	1.196 (2)	C15—C16	1.415 (3)
O2—C4	1.334 (2)	C15—H15A	0.9900
O2—C5	1.459 (2)	C16—H16A	0.9900
O3—C7	1.203 (2)	C21—C25	1.405 (3)
O4—C7	1.336 (2)	C21—C22	1.409 (3)
O4—C8	1.470 (2)	C21—H21A	0.9900
C1—C11	1.536 (3)	C22—C23	1.416 (3)
C1—C7	1.538 (3)	C22—H22A	0.9900
C1—C4	1.541 (3)	C23—C24	1.408 (3)
C1—C2	1.543 (3)	C23—H23A	0.9900
C2—C3	1.522 (3)	C24—C25	1.418 (3)
C2—H2A	0.9800	C24—H24A	0.9900
C2—H2B	0.9800	C25—H25A	0.9900
C3—H3A	0.9700	P1—F4'	1.508 (5)
C3—H3B	0.9700	P1—F3	1.510 (6)
C3—H3C	0.9700	P1—F5	1.523 (12)
C5—C6	1.486 (3)	P1—F2	1.538 (5)
C5—H5A	0.9800	P1—F1'	1.541 (4)
C5—H5B	0.9800	P1—F6	1.544 (11)
C6—H6A	0.9700	P1—F2'	1.582 (5)
C6—H6B	0.9700	P1—F4	1.586 (6)
C6—H6C	0.9700	P1—F1	1.586 (4)
C8—C9	1.496 (3)	P1—F6'	1.589 (13)
C8—H8A	0.9800	P1—F3'	1.590 (6)
C8—H8B	0.9800	P1—F5'	1.612 (14)
			
C21—Fe1—C24	67.56 (9)	C12—C13—Fe1	70.81 (11)
C21—Fe1—C25	40.06 (9)	C14—C13—H13A	119.0
C24—Fe1—C25	40.45 (9)	C12—C13—H13A	119.0
C21—Fe1—C23	67.69 (9)	Fe1—C13—H13A	119.0
C24—Fe1—C23	40.14 (9)	C15—C14—C13	119.4 (2)
C25—Fe1—C23	67.79 (9)	C15—C14—Fe1	70.05 (12)
C21—Fe1—C22	40.07 (9)	C13—C14—Fe1	70.18 (12)
C24—Fe1—C22	67.35 (9)	C15—C14—H14A	119.4
C25—Fe1—C22	67.37 (9)	C13—C14—H14A	119.4
C23—Fe1—C22	40.24 (9)	Fe1—C14—H14A	119.4
C21—Fe1—C15	128.85 (9)	C14—C15—C16	120.4 (2)
C24—Fe1—C15	106.60 (9)	C14—C15—Fe1	70.53 (12)
C25—Fe1—C15	101.40 (9)	C16—C15—Fe1	70.08 (11)
C23—Fe1—C15	140.10 (9)	C14—C15—H15A	118.8
C22—Fe1—C15	168.30 (9)	C16—C15—H15A	118.8
C21—Fe1—C16	106.85 (9)	Fe1—C15—H15A	118.8
C24—Fe1—C16	134.61 (9)	C11—C16—C15	120.80 (19)
C25—Fe1—C16	105.35 (9)	C11—C16—Fe1	72.02 (11)
C23—Fe1—C16	173.11 (9)	C15—C16—Fe1	70.00 (12)
C22—Fe1—C16	137.62 (9)	C11—C16—H16A	118.8
C15—Fe1—C16	39.93 (8)	C15—C16—H16A	118.8
C21—Fe1—C13	158.75 (9)	Fe1—C16—H16A	118.8
C24—Fe1—C13	116.55 (9)	C25—C21—C22	108.4 (2)
C25—Fe1—C13	154.37 (9)	C25—C21—Fe1	70.08 (12)
C23—Fe1—C13	101.40 (9)	C22—C21—Fe1	70.47 (12)
C22—Fe1—C13	120.09 (9)	C25—C21—H21A	125.8
C15—Fe1—C13	71.43 (9)	C22—C21—H21A	125.8
C16—Fe1—C13	85.11 (8)	Fe1—C21—H21A	125.8
C21—Fe1—C14	160.63 (9)	C21—C22—C23	108.0 (2)
C24—Fe1—C14	98.94 (9)	C21—C22—Fe1	69.46 (12)
C25—Fe1—C14	120.80 (9)	C23—C22—Fe1	69.50 (12)
C23—Fe1—C14	111.53 (9)	C21—C22—H22A	126.0
C22—Fe1—C14	148.93 (9)	C23—C22—H22A	126.0
C15—Fe1—C14	39.42 (9)	Fe1—C22—H22A	126.0
C16—Fe1—C14	72.10 (8)	C24—C23—C22	107.7 (2)
C13—Fe1—C14	39.53 (8)	C24—C23—Fe1	69.82 (12)
C21—Fe1—C12	127.06 (8)	C22—C23—Fe1	70.26 (12)
C24—Fe1—C12	149.64 (9)	C24—C23—H23A	126.1
C25—Fe1—C12	166.15 (8)	C22—C23—H23A	126.1
C23—Fe1—C12	115.23 (9)	Fe1—C23—H23A	126.1
C22—Fe1—C12	105.62 (8)	C23—C24—C25	108.2 (2)
C15—Fe1—C12	84.63 (8)	C23—C24—Fe1	70.05 (12)
C16—Fe1—C12	71.27 (8)	C25—C24—Fe1	69.87 (12)
C13—Fe1—C12	39.44 (8)	C23—C24—H24A	125.9
C14—Fe1—C12	71.60 (8)	C25—C24—H24A	125.9
C21—Fe1—C11	106.11 (8)	Fe1—C24—H24A	125.9
C24—Fe1—C11	170.92 (8)	C21—C25—C24	107.7 (2)
C25—Fe1—C11	130.58 (8)	C21—C25—Fe1	69.87 (12)
C23—Fe1—C11	144.91 (9)	C24—C25—Fe1	69.68 (12)
C22—Fe1—C11	112.43 (8)	C21—C25—H25A	126.2
C15—Fe1—C11	71.85 (8)	C24—C25—H25A	126.2
C16—Fe1—C11	39.38 (7)	Fe1—C25—H25A	126.2
C13—Fe1—C11	71.79 (8)	F4'—P1—F3	118.9 (5)
C14—Fe1—C11	85.47 (8)	F4'—P1—F5	95.7 (6)
C12—Fe1—C11	39.38 (7)	F3—P1—F5	87.7 (8)
C4—O2—C5	116.62 (16)	F4'—P1—F2	146.6 (5)
C7—O4—C8	116.15 (16)	F3—P1—F2	94.2 (4)
C11—C1—C7	110.06 (15)	F5—P1—F2	79.9 (7)
C11—C1—C4	108.78 (15)	F4'—P1—F1'	94.2 (4)
C7—C1—C4	107.62 (15)	F3—P1—F1'	145.1 (5)
C11—C1—C2	113.56 (16)	F5—P1—F1'	100.5 (8)
C7—C1—C2	108.40 (16)	F2—P1—F1'	54.9 (3)
C4—C1—C2	108.24 (16)	F4'—P1—F6	91.8 (7)
C3—C2—C1	114.76 (17)	F3—P1—F6	80.5 (6)
C3—C2—H2A	108.6	F5—P1—F6	168.0 (8)
C1—C2—H2A	108.6	F2—P1—F6	98.8 (6)
C3—C2—H2B	108.6	F1'—P1—F6	88.2 (6)
C1—C2—H2B	108.6	F4'—P1—F2'	174.9 (4)
H2A—C2—H2B	107.6	F3—P1—F2'	57.2 (4)
C2—C3—H3A	109.5	F5—P1—F2'	87.5 (6)
C2—C3—H3B	109.5	F2—P1—F2'	37.9 (3)
H3A—C3—H3B	109.5	F1'—P1—F2'	89.1 (4)
C2—C3—H3C	109.5	F6—P1—F2'	84.4 (7)
H3A—C3—H3C	109.5	F4'—P1—F4	29.7 (3)
H3B—C3—H3C	109.5	F3—P1—F4	89.2 (4)
O1—C4—O2	124.96 (18)	F5—P1—F4	96.3 (7)
O1—C4—C1	125.52 (18)	F2—P1—F4	174.8 (3)
O2—C4—C1	109.49 (16)	F1'—P1—F4	122.9 (4)
O2—C5—C6	110.53 (19)	F6—P1—F4	85.6 (7)
O2—C5—H5A	109.5	F2'—P1—F4	146.1 (4)
C6—C5—H5A	109.5	F4'—P1—F1	56.3 (3)
O2—C5—H5B	109.5	F3—P1—F1	173.4 (4)
C6—C5—H5B	109.5	F5—P1—F1	88.5 (7)
H5A—C5—H5B	108.1	F2—P1—F1	90.4 (4)
C5—C6—H6A	109.5	F1'—P1—F1	41.1 (3)
C5—C6—H6B	109.5	F6—P1—F1	103.5 (6)
H6A—C6—H6B	109.5	F2'—P1—F1	127.9 (4)
C5—C6—H6C	109.5	F4—P1—F1	85.9 (3)
H6A—C6—H6C	109.5	F4'—P1—F6'	88.7 (6)
H6B—C6—H6C	109.5	F3—P1—F6'	93.0 (6)
O3—C7—O4	124.71 (18)	F5—P1—F6'	174.6 (8)
O3—C7—C1	123.51 (18)	F2—P1—F6'	94.7 (6)
O4—C7—C1	111.77 (16)	F1'—P1—F6'	75.9 (6)
O4—C8—C9	110.54 (18)	F6—P1—F6'	12.9 (10)
O4—C8—H8A	109.5	F2'—P1—F6'	88.3 (6)
C9—C8—H8A	109.5	F4—P1—F6'	89.0 (6)
O4—C8—H8B	109.5	F1—P1—F6'	91.3 (6)
C9—C8—H8B	109.5	F4'—P1—F3'	89.7 (4)
H8A—C8—H8B	108.1	F3—P1—F3'	30.1 (4)
C8—C9—H9A	109.5	F5—P1—F3'	82.8 (8)
C8—C9—H9B	109.5	F2—P1—F3'	122.1 (4)
H9A—C9—H9B	109.5	F1'—P1—F3'	174.5 (4)
C8—C9—H9C	109.5	F6—P1—F3'	87.9 (6)
H9A—C9—H9C	109.5	F2'—P1—F3'	86.8 (4)
H9B—C9—H9C	109.5	F4—P1—F3'	60.5 (3)
C16—C11—C12	117.94 (18)	F1—P1—F3'	143.9 (4)
C16—C11—C1	122.20 (17)	F6'—P1—F3'	100.4 (6)
C12—C11—C1	119.85 (17)	F4'—P1—F5'	84.6 (5)
C16—C11—Fe1	68.60 (11)	F3—P1—F5'	98.0 (7)
C12—C11—Fe1	69.27 (11)	F5—P1—F5'	12.5 (10)
C1—C11—Fe1	133.56 (12)	F2—P1—F5'	86.2 (6)
C13—C12—C11	121.08 (19)	F1'—P1—F5'	95.7 (7)
C13—C12—Fe1	69.75 (12)	F6—P1—F5'	174.9 (9)
C11—C12—Fe1	71.34 (11)	F2'—P1—F5'	99.0 (5)
C13—C12—H12A	118.5	F4—P1—F5'	89.5 (6)
C11—C12—H12A	118.5	F1—P1—F5'	77.6 (7)
Fe1—C12—H12A	118.5	F6'—P1—F5'	168.9 (8)
C14—C13—C12	120.31 (19)	F3'—P1—F5'	88.4 (8)
C14—C13—Fe1	70.29 (12)		
